# Efficacy of gene therapy-delivered cytosine deaminase is determined by enzymatic activity but not expression

**DOI:** 10.1038/sj.bjc.6603624

**Published:** 2007-02-20

**Authors:** L Dubois, T Dresselaers, W Landuyt, K Paesmans, A Mengesha, B G Wouters, P Van Hecke, J Theys, P Lambin

**Affiliations:** 1 Department of Radiation Oncology (Maastro Lab), GROW Research Institute, University of Maastricht, UNS 50/23, PO Box 616, 6200 MD, Maastricht, The Netherlands; 2 Biomedical NMR Unit, K.U. Leuven, Herestraat 49, PO Box 803, 3000 Leuven, Belgium; 3 Laboratory of Experimental Oncology/Radiobiology, K.U. Leuven, Gasthuisberg-CDG 8th floor, Herestraat 49, 3000 Leuven, Belgium

**Keywords:** ^19^F-MRS, *Salmonella typhimurium*, gene therapy, cytosine deaminase, xenograft human tumour

## Abstract

The potential utility of tumour-selective 5-fluorouracil treatment using attenuated *Salmonella serovar typhimurium* recombinant for cytosine deaminase (TAPET-CD) has been documented in experimental settings. The present data demonstrate that *in vivo* ^19^F-magnetic resonance spectroscopy measurements allow the outcome prediction of this prokaryotic-based therapy, demonstrating the necessity of non-invasive real-time imaging techniques for treatment monitoring.

Positron emission tomography and (nuclear) magnetic resonance imaging (MRI) have been introduced to evaluate the biological characteristics of tumours and to monitor the efficacy of treatment on an individual basis. Fluor-containing chemotherapeutics, such as 5-fluorouracil (5-FU) and its metabolites, can be detected non-invasively in tumour and liver tissues using ^19^F-magnetic resonance spectroscopy (^19^F-MRS). Preclinical animal investigations and patient studies suggest that trapping of 5-FU in the tumour correlates with response and may provide prognostic information on treatment outcome (Wolf *et al*, 1990; Schlemmer *et al*, 1999; Martino *et al*, 2005).

Ample data exist on the use of the cytosine deaminase (CD) enzyme, which converts the nontoxic 5-fluorocytosine (5-FC) into the cytostatic 5-FU, in a number of experimental settings. Among these, we and others have demonstrated that recombinant bacteria such as *Clostridium* and attenuated *Salmonella*, can be used to transfer CD selectively to the rodent tumour microenvironment, resulting in antitumour effects (Minton *et al*, 1995; Theys *et al*, 2001; King *et al*, 2002; Mei *et al*, 2002). In addition, we reported the feasibility of ^19^F-MRS to assess non-invasively the intratumour conversion of 5-FC into 5-FU mediated by CD-recombinant *Salmonella* (Dresselaers *et al*, 2003).

The aim of the present study was to investigate the value of *in vivo* ^19^F-MRS to predict the success of the TAPET-CD/5-FC cancer therapy system and to evaluate it as a tool to allow individualised treatment.

## MATERIALS AND METHODS

HCT116 human colorectal carcinoma cells were xenografted (1.5 × 10^6^ cells per 100 *μ*l) subcutaneously at the lower flank of 8- to 10-week-old female NMRI nu/nu mice. Treatment and ^19^F-MRS measurements started when tumour volumes reached >300 mm^3^. Tumours were measured in three orthogonal dimensions with a calliper and volumes were calculated according to the formula *ABC* × *π*/6.

TAPET-CD (VION Pharmaceuticals Inc., New Haven, CT, USA), in detail described by Zheng *et al* (2000), was cultured as described previously (Mei *et al*, 2002) and 2.0 × 10^6^ bacteria were injected intravenously (tail vein). Six days after TAPET-CD administration, 5-FC was daily administered intraperitoneally in one group of animals (*n*=12), whereas a control group (*n*=4) received saline according to the same schedule.

To determine the intratumour conversion activity of the TAPET-CD, *in vivo* ^19^F-MRS experiments were performed in a 4.7 T BIOSPEC horizontal magnet (Bruker), equipped with a double-tuned (^19^F-^1^H) surface coil of 1 cm diameter, as described previously (Dresselaers *et al*, 2003). ^19^F-MRS measurements were performed four times on each animal, starting 6 days after TAPET-CD injection and then every sixth or seventh day. An additional 5-FC injection was given immediately before the ^19^F-MRS measurements.

At the end of the follow-up period, animals were killed, tumours excised and homogenates were made to quantify bacterial colonisation, presence (Western blotting) and activity (thin-layer chromatography (TLC)) of CD, according to Theys *et al* (2001). Briefly, Western blotting was carried out by loading 25 *μ*g of tumour cell extracts on the gel. Anti-CD monoclonal antibody (MTM Laboratories Inc., Westborough, MA, USA) in a 1 : 5000 dilution was used as primary antibody. After overnight incubation of the sonicated lysates with 5-FC (10 mg ml^−1^), the conversion into 5-FU was analysed by TLC on silica gel plates eluted with butanol. 5-Fluorocytosine and 5-FU were loaded as reference. Histological evaluation of hypoxia and necrosis was performed on paraffin-embedded tumour pieces after administration of the exogenous hypoxia marker pimonidazole. Staining and evaluation of the sections were performed as described previously (Dubois *et al*, 2004).

*In vitro* ^19^F-MRS analysis was performed on perchloric acid extracts using a AMX360 (8.4 T) spectrometer (Bruker, Karlsruhe, Germany; Dresselaers *et al*, 2003).

All animal experiments were in agreement with national guidelines, approved by the Animal Ethics Committee of the University ‘KU Leuven’, Belgium, and procedures were according to the guidelines defined by the UKCCCR (Workman *et al*, 1998).

## RESULTS

**Figure 1:**
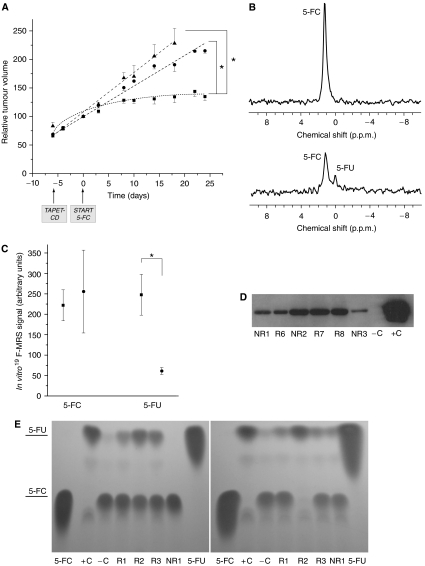
(**A**) Growth delay for ‘responder’ (9/12; ▪) and ‘nonresponder’ (3/12; •) animals. Sham saline-treated animals are indicated with ▴. Animals in which a 5-FU signal could be detected using *in vivo* ^19^F-MRS during the follow-up period were marked as ‘responders’, the others as ‘nonresponders’. The 100% value represents the tumour volume at day 0 (ie volume at the start of 5-FC administration). Statistical significance at *P*<0.001 using linear regression analysis is indicated (^*^). Data are shown as mean±s.e.m. (**B**) Representative spectra (13 min/spectrum: TR=0.75 s; NA=1024; LB=6 Hz; zerofilling to 4096 points) of a ‘nonresponder’ (top) and a ‘responder’ (bottom) animal. (**C**) Quantification of the amount of 5-FC and 5-FU using *in vitro* ^19^F-MRS on perchloric acid tumour extracts of ‘responders’ (▪) and ‘nonresponders’ (•). Statistical significance at *P*<0.05 using a nonparametric Mann–Whitney *U* test is indicated (^*^). Data are shown as mean±s.e.m. (**D**–**E**) Western blotting and thin-layer chromatography (TLC) for CD expression and activity respectively in tumour homogenates from HCT116 xenograft In NMRI nu/nu mice injected with TAPET-CD. TLC was done before (**E** left) and after (**E** right) adding Luria–bertani medium to the tumour extracts with overnight incubation. ‘Responders’ are indicated with R, ‘nonresponders’ with NR. The +C indicates a sample of bacterial lysate from *in vitro* growing TAPET-CD and represent the positive control. −C represents the negative control, that is, bacterial lysate from the *in vitro* growing PC0698 strain, which does not encode CD.

^19^F-MRS measurements were performed repeatedly on every animal starting at 6 days after TAPET-CD administration and immediately after the 5-FC injection. These spectroscopy analyses allowed us to evaluate the *in vivo* intratumour conversion of 5-FC into 5-FU, which resulted from TAPET-CD activity. Animals in which a 5-FU signal could be detected during the follow-up period were marked as ‘responders’ (9 out of 12), the others as ‘non-responders’ (3 out of 12). Linear regression analysis showed a significant tumour growth delay for the ‘responders’ compared to the ‘non-responders’ (29.00 days; *β*=5.72; *P*<0.001) and control saline-treated group (29.83 days; *β*=3.32; *P*<0.001), as witnessed from the interaction between time and group (Figure 1A). Representative spectra of ‘responder’ and ‘non-responder’ animals are shown in Figure 1B. The final *in vivo* measurements were subsequently confirmed by quantification of the 5-FU amount using *in vitro* ^19^F-MRS analyses. Whereas equal amounts of 5-FC were found, significant differences in 5-FU amount existed between ‘responders’ and ‘non-responders’ (*P*=0.044) (Figure 1C). This was not caused by a different level of colonisation because the number of viable bacteria present in the tumours was not significantly different in both groups (equally high colonisation, >10^9^ cfu g^−1^ tumour tissue). In addition, Western blotting on tumour extracts did not reveal differences in CD protein expression (Figure 1D). To exclude presence of mutations within the CD protein, lysates were made from bacteria isolated out of tumours from both ‘responders’ and ‘non-responders’. TLC experiments with these bacterial lysates demonstrated conversion of 5-FC to 5-FU in all cases, indicating a fully functional CD protein (data not shown). However, the ability for conversion of 5-FC into 5-FU, as determined by TLC experiments on tumour extracts, could only be shown in the ‘responders’ and not in the ‘non-responders’ group (Figure 1E left). This corresponds to the very small amount of 5-FU detected in the ‘non-responder animals’ using the more sensitive *in vitro* ^19^F-MRS (Figure 1C). Interestingly, the ‘no-conversion phenomenon’ in the ‘non-responder’ animals could be reversed by adding nutrients (Luria–Bertani medium) to the tumour extracts and allowing the mixture incubate overnight before making sonicated lysates (Figure 1E, right).

## DISCUSSION

The present study shows that the dynamic 5-FC/5-FU conversion in the human colorectal HCT116 tumour xenograft following systemic injection of TAPET-CD and 5-FC can be monitored using *in vivo* ^19^F-MRS. More importantly, we demonstrate the need for sequential non-invasive ^19^F-MRS imaging as a tool for TAPET-CD/5-FC therapy prediction. Given the fact that a pilot clinical trial with TAPET-CD, in which two out of three refractory cancer patients maintained stable disease when functional CD was produced within the tumour (Nemunaitis *et al*, 2003) have already started, our results point to the importance of not only monitoring tumour colonisation but also CD functionality.

Tumours in which a 5-FU signal could be detected using *in vivo* ^19^F-MRS during the follow-up period were marked as ‘responders’, the others were marked ‘non-responders’. Despite similar TAPET-CD colonisation levels in all tumours, growth delay assays showed a significant difference in success of the TAPET-CD/5-FC therapy for the ‘responders’ *vs* ‘non-responders’ and the control saline-treated animals. *In vitro* ^19^F-MRS analysis performed on the perchloric acid extracts of the resected tumours taken at the end of the follow-up period, confirmed the *in vivo* ^19^F-MRS 5-FC/5-FU measurements. Intriguingly, Western blotting did not reveal a difference in CD protein expression between the samples of ‘responders’ and ‘non-responders,’ and TLC analysis on bacterial lysates confirmed the CD functionality in all cases. This indicates that in the TAPET-CD present in the tumour, the CD gene was not selectively lost. In other words, expression of CD by recombinant bacteria in the tumour is not enough to establish an antitumour effect when combined with 5-FC administration. Kamm *et al* (1996) have argued that differences in 5-FU levels could be explained by morphological heterogeneity in tumours. However, we did not find histological differences in the amount of hypoxia/necrosis between the tumours of ‘responders’ and ‘non-responders’ nor in the levels of 5-FC detected in the tumours via ^19^F-MRS (both *in vitro* and *in vivo*). These results showed that prodrug diffusion to the bacterial colonisation sites in both groups is not impaired. As no differences were seen in bacterial colonisation and CD protein expression, but significance was obtained in growth delay between ‘responders’ and ‘non-responders’, we reasoned that the observed difference was probably owing to either the presence of an inhibitory factor in the tumours of the ‘non-responders’, or to the lack of factors needed for CD enzyme activity. The catalytic activity of CD is shown to be dependent on Fe^2+^ or other divalent metal ions that function as cofactor. Although it might be possible that these cofactors are present in varying amounts among tumours, this appeared not to be limiting in our study (data not shown). Alternatively, low intratumoural pH or high endogenous cytosine concentrations that compete with 5-FC for the active site might reduce CD activity. However, as TLC experiments revealed that adding fresh growth medium to the tumour extracts of non-responders resulted in 5-FC/5-FU conversion, our data point towards the absence of yet other necessary factors. From our results, it is apparent that in a subgroup of tumours, the absence of these necessary nutrients for efficient CD enzymatic activity can result in a lack of antitumour efficacy. Therefore, our results stress the importance of non-invasive real-time imaging tools as ^19^F-MRS for individualised treatment as it allows the prediction of antitumour efficacy when using TAPET-CD/5-FC therapy or related treatments.
